# Exploring gaps, biases, and research priorities in the evidence for reptile conservation actions

**DOI:** 10.1111/cobi.70073

**Published:** 2025-05-31

**Authors:** Oliver Speight, William H. Morgan, Thomas B. White, Katie A. Sainsbury, Amos Bouskila, Guy Rotem, Rebecca K. Smith, William J. Sutherland, Maggie J. Watson, Alec P. Christie

**Affiliations:** ^1^ Department of Zoology University of Cambridge Cambridge UK; ^2^ Department of Biology & Leverhulme Centre for Nature Recovery University of Oxford Oxford UK; ^3^ The Biodiversity Consultancy Cambridge UK; ^4^ Faculty of Kinesiology, Sport, and Recreation University of Alberta Edmonton Alberta Canada; ^5^ Department of Life Sciences Ben‐Gurion University of the Negev Beersheba Israel; ^6^ Bird Group Natural History Museum Tring UK; ^7^ Downing College Cambridge UK; ^8^ Centre for Environmental Policy Imperial College London London UK

**Keywords:** biodiversity, conservation, evidence‐based, evidence gaps, evidence‐informed, literature bias, reptiles, research bias, research gaps, acción de conservación, biodiversidad, conservación basada en evidencias, conservación de reptiles, conservación informada por evidencias, prácticas basadas en evidencias, vacíos en la investigación, **关键词**: 基于证据的保护, 有实证依据的保护, 保护行动, 爬行动物保护, 基于证据的实践, 生物多样性, 研究空缺

## Abstract

With over 21% of reptile species threatened with extinction, there is an urgent need to ensure conservation actions to protect and restore populations are informed by relevant, reliable evidence. We examined the geographic and taxonomic distribution of 707 studies that tested the effects of actions to conserve reptiles synthesized in Conservation Evidence's Reptile Conservation synopsis. More studies were conducted in countries with higher gross domestic product per capita, more reptile species, and higher proportions of threatened reptile species. Studies were clustered in the United States (43%) and Australia (15%), and no studies were conducted in large parts of Southeast Asia, South America, and sub‐Saharan Africa. Taxonomically, 47% of 90 reptile families (mostly Squamata) were not studied at all. Although Squamata and Testudines species featured in approximately 50% of studies, 7 of the 10 most‐studied reptiles (constituting 36% of studies) were turtles or tortoises, and there were significantly more studies per species on Testudines than Squamata. There were also significantly more studies on species: classified as least concern (as opposed to all other International Union for Conservation of Nature categories apart from near threatened); not categorized as endemic or insular; with more Wikipedia page views; and lacking data on venomousness. There was no significant relationship between the number of studies and the evolutionary distinctiveness or body mass of species. Our results highlight pressing evidence needs, particularly for underrepresented regions and threatened and data‐deficient species (e.g., evolutionarily distinct and globally endangered reptiles in South America, sub‐Saharan Africa, and Southeast Asia). To overcome evidence gaps and a lack of basic ecological data, future work should explore how the effects of actions transfer across taxa and regions. We call for greater efforts to coordinate and increase testing and reporting in a strategic manner to inform more effective and efficient conservation actions globally.

## Abstract (es)

Análisis de los vacíos, sesgos y prioridades de investigación en la evidencia de las acciones de conservación para reptiles

**Resumen:**

Con más del 21% de las especies de reptiles en peligro de extinción, existe una necesidad urgente de garantizar que las acciones de conservación para proteger y restaurar las poblaciones se basen en pruebas pertinentes y fiables. Examinamos la distribución geográfica y taxonómica de 707 estudios que probaron los efectos de las acciones para conservar los reptiles sintetizados en la sinopsis de Conservación de Reptiles de Conservation Evidence. Se realizaron más estudios en países con mayor producto interino bruto per cápita, más especies de reptiles y mayor proporción de especies de reptiles amenazadas. Los estudios se concentraron en los Estados Unidos (43%) y Australia (15%), y no se realizaron estudios en grandes zonas del sudeste asiático, Sudamérica y África subsahariana. Desde el punto de vista taxonómico, el 46% de las 92 familias (en su mayoría Squamata) no se estudiaron en absoluto. Aunque las especies de Squamata y Testudines figuraban en aproximadamente el 50% de los estudios, 7 de los 10 reptiles más estudiados eran tortugas terrestres o de las Galápagos (36% de los estudios), y había muchos más estudios por especie sobre Testudines que sobre Squamata. También hubo un número significativamente mayor de estudios sobre especies clasificadas como menos preocupantes (frente a todas las demás categorías de la Unión Internacional para la Conservación de la Naturaleza, salvo la de casi amenazadas); no categorizadas como endémicas o insulares; con más páginas vistas en Wikipedia; y que carecían de datos sobre toxicidad. No observamos ninguna relación significativa entre el número de estudios y el carácter distintivo evolutivo o la masa corporal de las especies. Nuestros resultados resaltan la urgente necesidad de pruebas, en particular para las regiones infrarrepresentadas y las especies amenazadas y sobre las que se carece de datos (por ejemplo, reptiles evolutivamente distintos y en peligro de extinción en Sudamérica, África subsahariana y el Sudeste Asiático). Para llenar los vacíos de pruebas y la falta de datos ecológicos básicos, los trabajos futuros deberían explorar cómo se transfieren los efectos de las acciones entre taxones y regiones. Pedimos que se redoblen los esfuerzos para coordinar y aumentar las pruebas y los informes de forma estratégica, con el fin de fundamentar acciones de conservación más eficaces y eficientes a escala mundial.

## 探究爬行动物保护行动证据中的空缺、偏倚和优先研究重点

**【摘要】** 目前已有超过21%的爬行动物物种濒临灭绝, 因此迫切需要确保保护和恢复种群的行动建立在相关的可靠证据之上。本研究分析了“保护证据(Conservation Evidence)”的《爬行动物保护概要》中总结的707项检验爬行动物保护行动效果的研究的地理分布和研究类群。我们发现, 在人均国内生产总值较高、爬行动物物种较多、受威胁爬行动物物种比例较高的国家开展的研究较多。研究主要集中在美国(43%)和澳大利亚(15%), 而在东南亚、南美洲和撒哈拉以南非洲的大部分地区没有开展相关研究。在类群方面, 爬行动物的92个科中有46%(主要是有鳞目)完全没有研究。尽管有鳞目和龟鳖目物种的研究占50%左右, 但在研究最多的10种爬行动物中, 有7种是海龟或陆龟(占研究的36%), 且龟鳖目每个物种得到的研究明显多于有鳞目。此外, 以下物种的研究也明显较多:被归为“无危”的物种(相对于《国际自然保护联盟》除“近危”以外所有濒危等级的物种), 非特有种和非岛屿种, 维基百科页面浏览量较高的物种, 以及缺乏毒性数据的物种。研究数量与物种的演化独特性或体重没有明显关系。我们的研究结果强调了迫切的证据需求, 尤其是对于代表性不足的地区以及受威胁和数据不足的物种(如南美洲、撒哈拉以南非洲和东南亚地区具有演化独特性且在全球范围内濒危的爬行动物)。为了解决证据空缺和基本生态数据缺乏的问题, 未来的工作应探究保护行动如何在不同类群和地区之间传递影响。我们呼吁加大力度, 以战略性的方式协调和增加对保护有效性的检验与报告, 来为全球更有效和高效的保护行动提供信息。**【翻译:胡怡思;审校:聂永刚】**

## INTRODUCTION

Global assessments of extinction risk suggest that 41% of amphibians, 25% of mammals, 14% of birds (IUCN, 2022), and 21% of reptiles (Cox et al., 2022) are threatened with extinction (i.e., are categorized as vulnerable, endangered, or critically endangered by the International Union for Conservation of Nature [IUCN] Red List [IUCN, 2022]). However, 15% of reptile species are also classified by the IUCN as data deficient (Cox et al., 2022), which, along with not evaluated species, are more likely to be threatened than those that have been assessed (Caetano et al., 2022; Gumbs et al., 2020). This suggests that reptiles are among the most threatened of the tetrapod groups. Major threats driving their relatively high extinction risk include changes in land use (impacts of agriculture, logging, and urbanization), introduction of invasive species, and climate change (Cox et al., 2022). These threats vary taxonomically and geographically, as does knowledge of their conservation status. According to the IUCN Red List of Threatened Species, the percentage of threatened species of Testudines (66%) and Crocodilia (48%) is higher than that of Squamata (17%), and the last Rhynchocephalia species is classified as least concern (IUCN, 2022). However, 15% of Squamata are also data deficient and thus likely to be threatened as well (Caetano et al., 2022; Gumbs et al., 2020), whereas only 4% of Testudines and no Crocodilia species are classified as data deficient. The highest number of threatened reptiles occurs in Southeast Asia, West Africa, Madagascar, the northern Andes, and the Caribbean; northern Europe and North America have the fewest (Cox et al., 2022).

Funding needs for biodiversity far exceed supply (Deutz et al., 2020). As such, maximizing the impact of these resources by ensuring conservation projects are as effective as possible is vital. One way to achieve this is through evidence‐based conservation, an approach that uses evidence (defined as any relevant data, information, knowledge, and wisdom used to assess an assumption related to a question of interest (Christie et al., 2023; Salafsky et al., 2022) to inform more effective conservation policies and practices (Sutherland et al., 2004). Summaries of relevant scientific evidence on the effectiveness of actions exist (Sutherland et al., 2019); however, resources, such as the Conservation Evidence database (www.conservationevidence.com), highlight that the evidence available often contains many gaps and biases in terms of the species, regions, and habitats covered (Christie et al., 2020, 2021; Junker et al., 2020; Taylor et al., 2019).

Given that preventing species extinctions is a major goal in conservation (Rounsevell et al., 2020), it is reasonable to expect biases in the evidence base toward threatened species and regions where extinction risks are higher. Biases toward more evolutionarily distinct species may also be seen as beneficial because by conserving these species, more phylogenetic diversity is conserved and it is less likely that evidence from other proxy species can be applied (Isaac et al., 2007; Jetz et al., 2014; Mace et al., 2003). Conversely, evidence biases toward so‐called charismatic species (i.e., popular species that are often used to raise money and awareness for conservation goals [Ducarme et al., 2013]) may be less beneficial for preventing species extinctions, particularly when these species are not threatened or evolutionarily distinct. The use of charismatic species as umbrella species (species whose conservation will aid other co‐occurring species [Roberge & Angelstam, 2004]) may be less problematic; however, reptiles are rarely used as umbrella species (Yang et al., 2023).

Studies on the evidence base for other taxa have generally failed to find positive biases toward more threatened species (Christie et al., 2021; Junker et al., 2020); instead, they show biases due to other factors. For example, more European Union LIFE scheme funding goes toward animals than plants (Adamo et al., 2022), more conservation research is focused on vertebrates than invertebrates (Donaldson et al., 2017), and more research focuses on mammals than other classes (Clark & May, 2002). Within groups of species, larger bodied species attract more conservation research (dos Santos et al., 2020; Tensen, 2018) and tend to be biased toward wealthier regions, where there tend to be fewer threatened species (Christie et al., 2020, 2021; Di Marco et al., 2017). For reptiles, in snake venom research, biases toward more hazardous species (often from the families Viperidae and Elapidae) have been detected, though whether these same trends are reflected in wider conservation research remains an open question (Avella et al., 2022).

We focused on the global evidence base for the effectiveness of reptile conservation actions, which has been compiled as part of the Conservation Evidence database (Sainsbury et al., 2021). We tested for geographic and taxonomic biases in the evidence base and explore whether any biases that exist reflect proxies of extinction risk, conservation priorities, and popularity of species. If research was allocated according to conservation needs, we expected that the evidence base would be biased toward the more biodiverse tropics, where more species are threatened (Cox et al., 2022; Roll et al., 2017), and toward orders with more threatened species (i.e., Crocodilia and Testudines [Cox et al., 2022]) and high levels of endemism.

## METHODS

### Identifying studies

To explore biases in the evidence base for reptile conservation, we extracted data from the Conservation Evidence database (specifically the Reptile Conservation synopsis [Sainsbury et al., 2021]), a freely accessible resource that provides summaries of studies that have quantitatively tested the effects of conservation interventions on biodiversity (Sutherland et al., 2019). The database represents the most comprehensive synthesis of the global evidence available for the effectiveness of reptile conservation actions and includes 707 studies (individual articles or reports [Sainsbury et al., 2021]) and metadata for each study (e.g., target species of each intervention tested and the study location). The database was compiled using subject‐wide evidence synthesis (Sutherland et al., 2019), which involved systematic searches for studies (by comprehensively screening all papers published) in certain English‐language journals and report series and in German‐ and Spanish‐language journals (*Herpetozoa* and *Revista de Biologia Tropical*, respectively) up to 2018. Details on specifics of the searches, including journals and years searched, inclusion and exclusion criteria, and protocols for extracting data, are in the Reptile Conservation synopsis (Sainsbury et al., 2021).

### Testing for geographic and taxonomic biases

To investigate geographic biases in the evidence base, we obtained the country name and geographic coordinates for each study from the Reptile Conservation synopsis (Sainsbury et al., 2021) and calculated the number of studies per country. We also collected data on 3 factors that might explain the number of studies per country: number of reptile species and proportion of threatened reptile species per country (Roll & Meiri, 2022), continent, and gross domestic product (GDP) per capita in the year the study was published (The World Bank, 2024). We used a country list from the World Bank (The World Bank, 2024) with data on GDP per capita for 208 countries in total where reptile species exist.

To investigate taxonomic biases, we calculated the number of studies per species (for all reptile species) from the Reptile Conservation synopsis (Sainsbury et al., 2021) and compared this number with 7 explanatory variables. Four explanatory variables covered proxies of extinction risk and conservation priorities: IUCN Red List category, insularity and endemism (yes or no), evolutionary distinctiveness (ED) score (The Zoological Society of London, 2020), and maximum body mass (grams). A further 3 explanatory variables covered taxonomy and proxies of popularity: taxonomic order, venomousness, and Wikipedia page views (from 2010 to 2020 derived with the *RSelenium* package [Harrison, 2022] and as used by Roll et al. [2016] and Willemen et al. [2015]). We obtained data on these factors for reptile species from Gumbs et al. (2024), the RepTraits database (Oskyrko et al., 2024), and SquamBase (Meiri, 2024). The ED score has not been calculated for species that are either extinct or extinct in the wild, so these species were not included in this part of the analyses (32 extinct and 2 extinct in the wild species). *Ctenotus fallens* also lacked an ED score. For other species, there was a lack of data on body mass (52 species), insularity and endemism (7), venomousness (1648 labeled unknown), and IUCN Red List assessment (479 labeled not evaluated). This left 10,386 species for our modeling data set. The ED scores, Wikipedia page views, and body mass were scaled by subtracting the mean and dividing by 2 SDs of each value (i.e., half *z* score standardization) to allow for direct comparisons between the variables following recommendations by Gelman et al. (2007) and Grueber et al. (2011). Because there was only one Rhynchocephalia species, we excluded this taxonomic order from our analyses. To visualize taxonomic biases at a family level, phylogenetic trees were created with ggtree (Yu et al., 2017) and phylogenies from Wiens et al. (2012) and Zheng and Wiens (2016).

### Statistical analyses

To evaluate geographic and taxonomic biases, we fitted generalized linear models (GLMs) with a negative binomial error structure (with log link function) with the package glmmTMB (Brooks et al., 2017). This error structure better accounted for zero inflation than a Poisson error structure from inspecting QQ plots and zero‐inflation tests produced using the DHARMa package (Hartig, 2022). We tested the assumption of the negative binomial model that the conditional means are not equal to the conditional variances with a likelihood ratio test (by comparing a Poisson model to the negative binomial model [UCLA, Statistical Consulting Group, 2024]) and found that for both global models (see below), the *χ* ^2^ was statistically significant (geographic: *χ* ^2^ = 1785.742, df = 5, *p* < 0.00001; taxonomic: *χ* ^2^ = 2253.063, df = 16, *p* < 0.00001), indicating that the assumption held and that the negative binomial model was more appropriate than the Poisson model. We did not find any clear patterns of problematic correlations between the explanatory variables used for each model, and Pearson's correlation coefficients for continuous variables were *r* < |0.34|.

For both geographic and taxonomic analyses, model selection was carried out by first specifying a global GLM. The global geographical GLM was:

(1) $$\begin{array}{ccc} & & \mathrm{number}\;\mathrm{of}\;\mathrm{studies}∼\mathrm{continent}+\mathrm{number}\;\mathrm{of}\;\mathrm{species}\\ & & +\;\mathrm{proportion}\;\mathrm{of}\;\mathrm{threatened}\;\mathrm{species}\;+\mathrm{GDP}\;\mathrm{per}\;\mathrm{capita}. \end{array}$$

The global taxonomic GLM was:

(2) $$\begin{array}{ccc} & & \mathrm{number}\;\mathrm{of}\;\mathrm{studies}∼\mathrm{IUCN}\;\mathrm{Red}\;\mathrm{List}\;\mathrm{category}+\mathrm{ED}\;\mathrm{score}\\ & & +\;\mathrm{Wikipedia}\;\mathrm{page}\;\mathrm{views}\;+\mathrm{taxonomic}\;\mathrm{order}+\mathrm{body}\;\mathrm{mass}\\ & & +\;\mathrm{venomous}+\mathrm{insular}\_\mathrm{endemic}. \end{array}$$

We used the Dredge function to consider the full set of models (including a null model). This resulted in a single top geographic model based on a difference in Akaike Information Criterion corrected for small sample sizes (ΔAIC_c_) with a threshold of 2 AIC_c_ units (see “RESULTS”). For taxonomic models, across the full total model set of 128 (Appendix S2), we found 3 top models that were of similar weights and had a ΔAIC_c_ threshold of 2AIC_c_ units (see “RESULTS”). We used the zero method to conduct model averaging across these 3 top models. A parameter estimate and an error of zero are substituted into those models when the given parameter is absent, and the parameter estimate is obtained by averaging over all models in the top model set (Burnham & Anderson, 2002). We used the zero method because we wanted to determine which explanatory variables had the strongest effect on the response variable (Grueber et al., 2011; Nakagawa & Freckleton, 2010). We also explored the importance of each explanatory variable based on relative importance, which takes the sum of model weights for all top models that include that variable and information on the number of top models in which the variable is included (Bartoń, 2024; Grueber et al., 2011). All data and code used for analyses are available from https://zenodo.org/doi/10.5281/zenodo.11282633 and were performed in R (R Core Team, 2023).

## RESULTS

**FIGURE 1:**
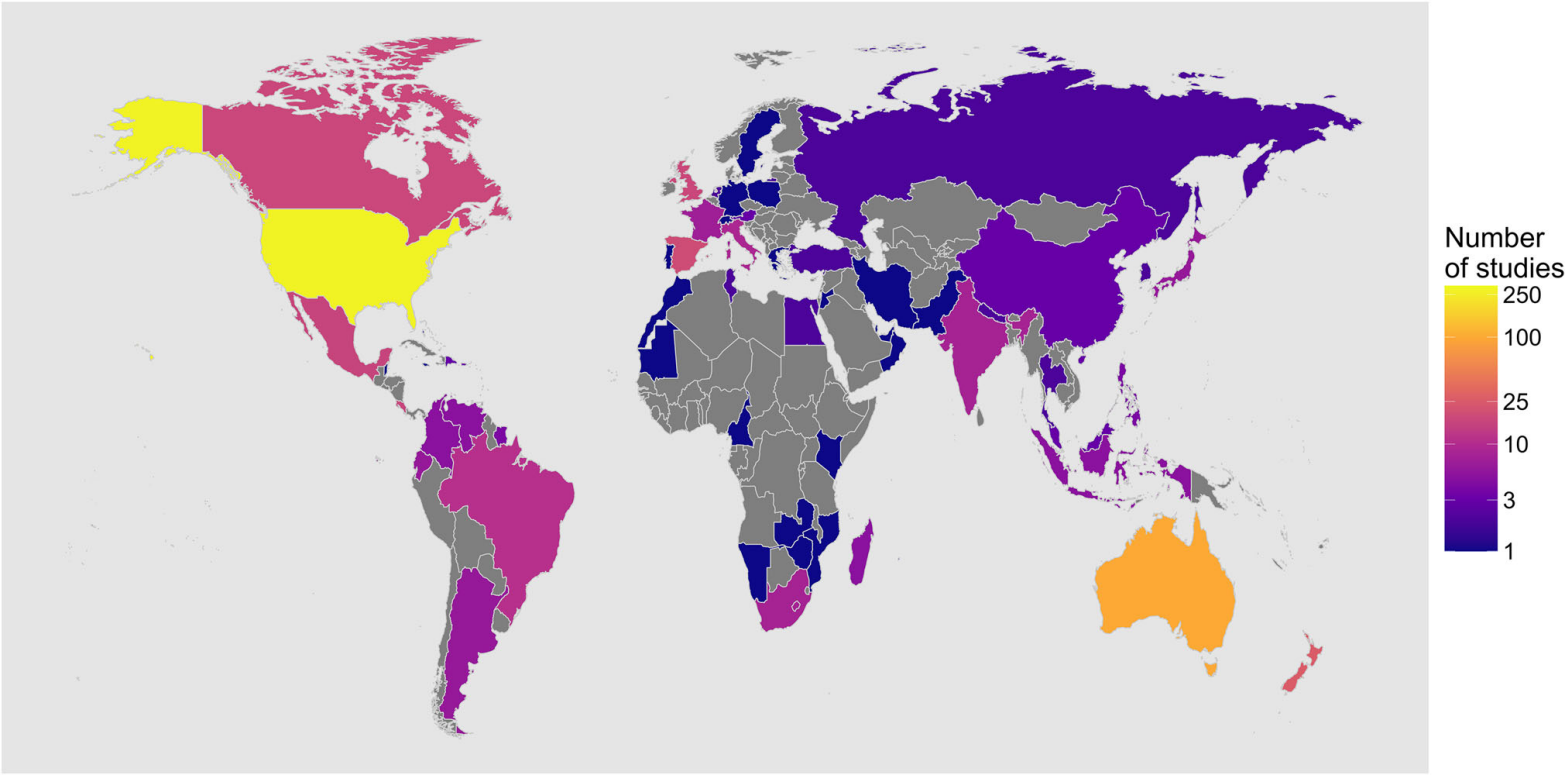
Number of studies that tested conservation actions on reptiles by country (gray, zero studies).

**FIGURE 2:**
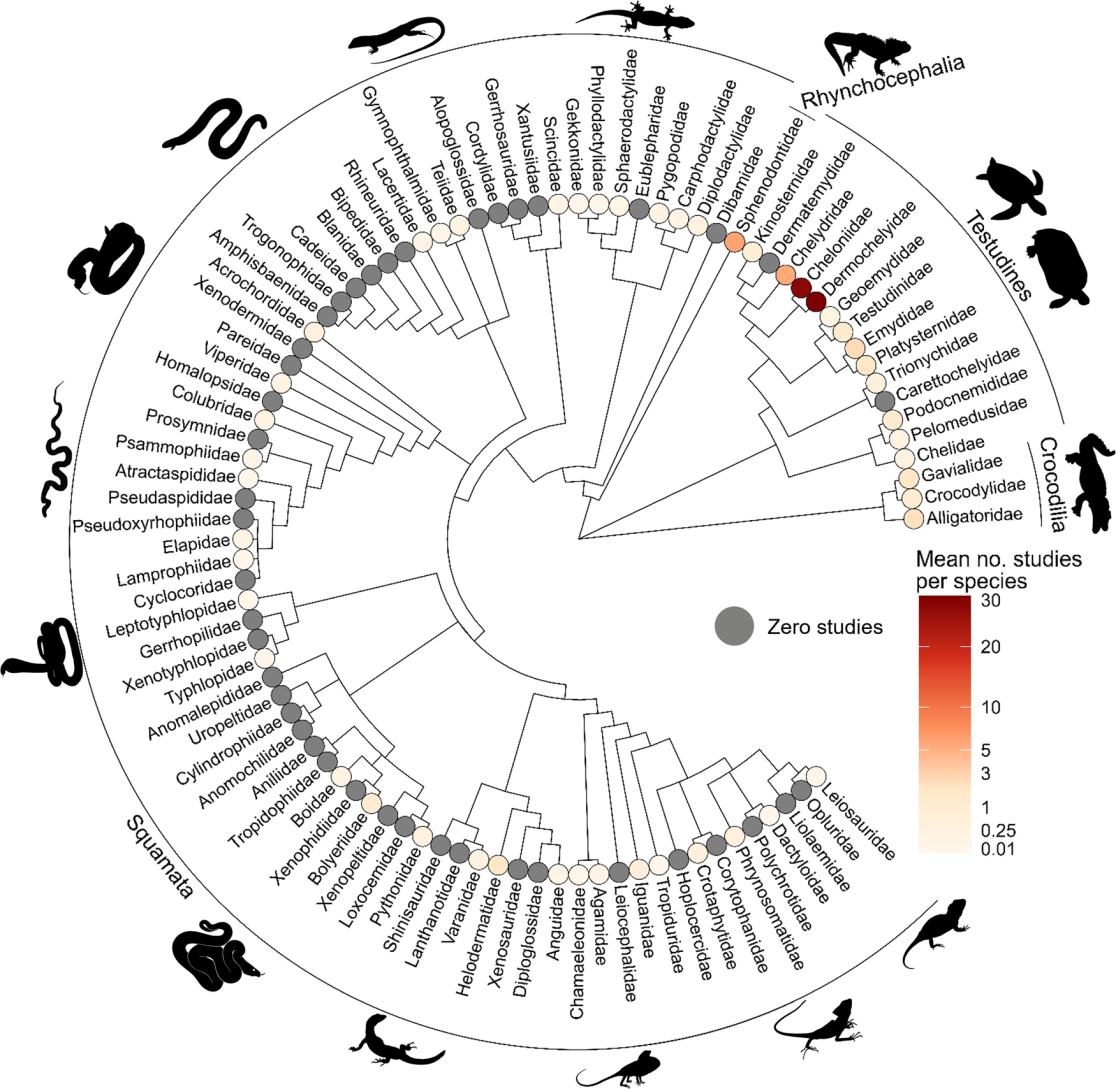
A phylogenetic tree of 90 reptile families showing the average number of studies that tested conservation actions on reptiles per species in each family (nodes). Families with higher numbers of studies per species have nodes in a darker red colour, whilst those with lower number have nodes in a paler shade. Based on phylogenies from Zheng and Wiens (2016) and Wiens et al. (2012). Families with a gray node were not featured in any study.

The Conservation Evidence database contained 707 studies on reptiles conducted in 64 countries (Figure 1) and for 508 species across 48 families (Figure 2).

### Geographic biases

Studies were not evenly distributed between countries. The 5 most represented countries were the United States (43% of studies, 277 studies) (Figure 1), Australia (15%, 100 studies), New Zealand (4%, 26 studies), Spain (3%, 19 studies), and the United Kingdom (3%, 17 studies). The best geographic GLM contained all explanatory variables except for continent, suggesting it was not a useful predictor of the number of studies per country. Countries with higher GDP per capita, more reptile species, and higher proportions of threatened species (in descending order of magnitude) tended to have more studies (number of species: *χ* ^2^ = 30.446, *p* < 0.001; proportion of threatened species: *χ* ^2^ = 7.043, *p* = 0.008; GDP per capita: *χ* ^2^ = 43.284, *p* < 0.001, df = 203) (Table 1).

**TABLE 1:** Summary statistics from the geographic generalized linear model predicting the number of studies that tested a reptile conservation intervention per country based on the explanatory variables gross domestic product per capita, number of reptile species, and proportion of reptile species threatened.

Model terms	Estimate	SE	*Z*	*p*	2.5% CI	97.5% CI	*χ* ^2^ ^a^	*χ* ^2^ df^a^	*χ* ^2^ *p* ^a^
Intercept	−0.248	0.167	−1.484	0.138	−0.576	0.080	–	–	–
Gross domestic product per capita (scaled^b^)	2.742	0.417	6.579	<0.001	1.925	3.559	43.284	1	<0.001
Number of reptile species (scaled^b^)	1.970	0.357	5.518	<0.001	1.270	2.670	30.446	1	<0.001
Proportion of reptile species threatened (scaled^b^)	0.851	0.321	2.654	0.008	0.222	1.479	7.043	1	0.008

^a^ Results from the analysis of deviance (type II Wald tests).

^b^ All explanatory variables were scaled by subtracting the mean and dividing by 2 SDs of each value (i.e., half *z* score standardization).

### Taxonomic biases

Of the 90 reptile families considered, 48 (53%) featured in at least one study, whilst the remaining 42 (47% families did not feature in any study (Figure 2, gray nodes). Squamata were most represented in the evidence base by total number of studies, albeit poorly relative to their high species richness (50% or 351 studies, 0.035 studies per species). Other taxonomic orders were studied better relative to their species richness: Testudines, 49% or 348 studies, 1.13 studies per species; Crocodilia, 5% or 36 studies, 1.50 studies per species; and Rhynchocephalia, 1% or 8 studies for its single species. Of the 10 most studied species, 7 were turtles and 2 were tortoises (the other was a snake) (Appendix S1), with Testudines featured in 36% of (254) studies. The 2 most studied families were Cheloniidae and Dermochelyidae (marine turtles), although Dermochelyidae was represented by only a single species (leatherback sea turtle [*Dermochelys coriacea*]).

The 3 best taxonomic models (ΔAIC_c_ < 2) all contained IUCN Red List category, Wikipedia page views, endemism, taxonomic order, and venomousness, but they differed in whether they contained ED score and body mass (Table 2). All models are described in Appendix S2. In the model that averaged across the 3 best models (Table 3), the most important predictors based on relative importance were endemism, taxonomic order, Wikipedia page views, IUCN Red List category, and venomousness (relative importance of 1 [Table 3]). Other predictors were relatively less important. The ED score and body mass were of 28% and 19% relative importance compared to the aforementioned variables (Table 3). The 95% confidence intervals for ED score and body mass also overlapped zero, indicating there was little evidence that these variables affected the number of studies per species (ED score, *z* = 0.374, *p* = 0.709; body mass, *z* = 0.048, *p* = 0.962) (Table 3).

**TABLE 2:** Summary of 3 individual taxonomic models predicting the number of studies that tested a reptile conservation intervention selected from a full set of 128 models^a^ based on having a difference in Akaike Information Criterion corrected for small sample sizes (ΔAIC_c_) of more than 2 AIC_c_ units versus other models.

Model 1						
Model term	Estimate	SE	*Z*	*p*	2.5% CI	97.5% CI
Intercept	−2.312	0.071	−32.449	<0.001	−2.452	−2.172
Insular or endemic (yes)	−0.477	0.146	−3.260	0.001	−0.764	−0.190
Order Testudines	2.064	0.240	8.594	<0.001	1.594	2.535
Order Crocodilia	0.856	0.724	1.182	0.237	−0.564	2.276
Wikipedia page views scaled	2.056	0.228	9.021	<0.001	1.609	2.503
RL: data deficient	−3.695	0.601	−6.144	<0.001	−4.874	−2.516
RL: not evaluated	−1.750	0.373	−4.696	<0.001	−2.480	−1.019
RL: near threatened	−0.461	0.251	−1.838	0.066	−0.954	0.031
RL: vulnerable	−0.598	0.241	−2.480	0.013	−1.070	−0.125
RL: endangered	−0.464	0.221	−2.099	0.036	−0.898	−0.031
RL: critically endangered	−0.602	0.295	−2.039	0.041	−1.181	−0.023
Venomous (yes)	−0.170	0.163	−1.046	0.296	−0.489	0.149
Venomous (unknown)	−1.273	0.213	−5.978	<0.001	−1.691	−0.856

Model 2						
Model term						
Intercept	−2.305	0.072	−32.203	<0.001	−2.446	−2.165
ED score scaled	−0.092	0.105	−0.876	0.381	−0.299	0.114
Insular or endemic (yes)	−0.474	0.146	−3.237	0.001	−0.760	−0.187
Order: Testudines	2.085	0.242	8.619	<0.001	1.611	2.559
Order: Crocodilia	0.857	0.723	1.185	0.236	−0.561	2.275
Wikipedia page views scaled	2.051	0.227	9.040	<0.001	1.606	2.496
RL: data deficient	−3.697	0.600	−6.159	<0.001	−4.873	−2.520
RL: not evaluated	−1.769	0.374	−4.731	<0.001	−2.502	−1.036
RL: near threatened	−0.462	0.251	−1.839	0.066	−0.955	0.030
RL: vulnerable	−0.593	0.241	−2.463	0.014	−1.065	−0.121
RL: endangered	−0.468	0.221	−2.117	0.034	−0.901	−0.035
RL: critically endangered	−0.585	0.295	−1.987	0.047	−1.163	−0.008
Venomous (yes)	−0.195	0.165	−1.182	0.237	−0.517	0.128
Venomous (unknown)	−1.302	0.215	−6.046	<0.001	−1.724	−0.880

Model 3						
Model term						
Intercept	−2.311	0.071	−32.338	<0.001	−2.451	−2.171
Maximum body mass scaled	0.011	0.104	0.109	0.913	−0.193	0.216
Insular or endemic (yes)	−0.478	0.147	−3.259	0.001	−0.765	−0.191
Order: Testudines	2.060	0.244	8.440	<0.001	1.581	2.538
Order: Crocodilia	0.807	0.850	0.950	0.342	−0.858	2.472
Wikipedia page views scaled	2.054	0.228	8.989	<0.001	1.606	2.502
RL: data deficient	−3.696	0.602	−6.144	<0.001	−4.875	−2.517
RL: not evaluated	−1.748	0.373	−4.691	<0.001	−2.479	−1.018
RL: near threatened	−0.461	0.251	−1.838	0.066	−0.953	0.031
RL: vulnerable	−0.601	0.243	−2.471	0.013	−1.079	−0.124
RL: endangered	−0.464	0.221	−2.098	0.036	−0.898	−0.030
RL: critically endangered	−0.603	0.295	−2.040	0.041	−1.182	−0.024
Venomous (yes)	−0.170	0.163	−1.046	0.296	−0.488	0.149
Venomous (unknown)	−1.273	0.213	−5.979	<0.001	−1.691	−0.856

Abbreviations: ED, evolutionary distinctiveness; RL, International Union for Conservation of Nature Red List category.

^a^ These models were derived from a global generalized linear model with the response variable of number of studies and the explanatory variables IUCN Red List category, evolutionary distinctiveness score, Wikipedia page views, taxonomic order, body mass, venomousness, and endemism. All models considered are presented in Appendix S2.

**TABLE 3:** Summary of final taxonomic model derived from a global generalized linear model with the response variable of number of studies and the explanatory variables International Union for Conservation of Nature Red List category, evolutionary distinctiveness score, Wikipedia page views, taxonomic order, body mass, venomousness, and endemism.

Model term	Est.	SE	Adjusted SE	*Z*	*p*	2.5% CI	97.5% CI	Rel.Imp.
Intercept	−2.310	0.071	0.071	32.327	<0.001	−2.450	−2.170	–
Insular or endemic (yes)	−0.476	0.146	0.146	3.253	0.001	−0.763	−0.189	1.000
Order: Testudines	2.069	0.242	0.242	8.562	<0.001	1.596	2.543	1.000
Order: Crocodilia	0.847	0.750	0.750	1.129	0.259	−0.624	2.317	
Wikipedia page views scaled	2.054	0.228	0.228	9.019	<0.001	1.608	2.501	1.000
RL: data deficient	−3.696	0.601	0.601	6.147	<0.001	−4.874	−2.517	1.000
RL: not evaluated	−1.755	0.373	0.373	4.703	<0.001	−2.486	−1.024	
RL: near threatened	−0.462	0.251	0.251	1.838	0.066	−0.954	0.031	
RL: vulnerable	−0.597	0.242	0.242	2.473	0.013	−1.071	−0.124	
RL: endangered	−0.465	0.221	0.221	2.103	0.035	−0.899	−0.032	
RL: critically endangered	−0.598	0.295	0.295	2.024	0.043	−1.176	−0.019	
Venomous (yes)	−0.177	0.164	0.164	1.082	0.279	−0.497	0.144	1.000
Venomous (unknown)	−1.281	0.214	0.214	5.986	<0.001	−1.701	−0.862	
ED score scaled	−0.026	0.070	0.070	0.374	0.709	−0.163	0.111	0.282
Maximum body mass scaled	0.002	0.046	0.046	0.048	0.962	−0.088	0.092	0.193

Abbreviations: ED, evolutionary distinctiveness; RL, International Union for Conservation of Nature Red List category.

*Note*: Estimates were obtained by model averaging with the zero method, with the top 3 models chosen by a difference in Akaike Information Criterion corrected for small sample sizes (ΔAIC_c_) of <2 (Table 2). All models considered are presented in Appendix S2.

Species with more Wikipedia page views tended to have more studies (*z* = 9.019, *p* < 0.001, 95% CIs >0) (Table 3), and Wikipedia page views were similar in magnitude to other predictors. In descending order, the species with the top 5 most page views were komodo dragon (*Varanus komodoensis*) (vulnerable), black mamba (*Dendroaspis polylepis*) (least concern), king cobra (*Ophiophagus Hannah*) (vulnerable), saltwater crocodile (*Crocodylus porosus*) (least concern), and cottonmouth snake (*Agkistrodon piscivorus*) (least concern) (100 most viewed species in Appendix S3). Venomous species did not have significantly more studies than nonvenomous species (z = 1.082, p = 0.279), and species lacking information on venomousness tended to have significantly fewer studies (*z* = 5.986, *p* < 0.001) (Table 3).

Conversely, species that were insular or endemic tended to have fewer studies (*z* = 3.253, *p* = 0.001), and there were significant differences between the number of studies for different taxonomic orders. There were significantly more studies for Testudines than Squamata (*z* = 8.562, *p* < 0.001), although there was no difference with Crocodilia (*z* = 1.129, *p* = 0.259) (Table 3). Species classified as least concern by the IUCN Red List had significantly more studies than data‐deficient and not evaluated species (*z* = 6.147 and 4.703, both *p* < 0.001) (Table 3), and marginally more than vulnerable, endangered, and critically endangered species (*z* = 2.024–2.473, *p* = 0.013–0.043) (Table 3), but not more than near Ithreatened species (*z* = 1.838, *p* = 0.066) (Table 3).

## DISCUSSION

### Drivers of gaps and biases

Many reptile species urgently need conservation action to address threatening processes and restore populations (Cox et al., 2022). Our investigation assessed the Conservation Evidence database, which has collated studies on the effectiveness of actions for reptile conservation, highlighting the coverage, biases, and gaps within the data available. We found, for example, that almost half of studies looked at the effects of actions on Testudines (turtles, terrapins, and tortoises)—a taxon of key conservation and public concern. However, there was no evidence on the effects of conservation actions for 47% of reptile families and we identified a major imbalance in which families are studied. Notably, there was a lack of studies for many Squamata (snakes, lizards, and amphisbaenians), despite 97% of all reptiles belonging to this order.

Countries with more studies tended to have a higher GDP per capita, higher numbers of reptile species, and higher proportions of threatened reptile species. This could be because research effort is being focused on important areas for reptile conservation or simply because it is more likely that research is conducted where more species occur. However, this pattern may also be largely driven by the high proportion of studies that were conducted in the United States and Australia (collectively representing 58% of studies). We have identified other significant geographic and taxonomic gaps and biases, which indicate that overall effort is not allocated in line with conservation priorities. First, there were more studies on species classified by the IUCN Red List as least concern than data deficient and not evaluated species, and marginally more than vulnerable, endangered, and critically endangered species. Second, there was no evidence to suggest more evolutionarily distinct species were studied more than others, despite the importance of distinctiveness in setting conservation priorities to protect phylogenetic and functional diversity (Isaac et al., 2007; Jetz et al., 2014; Mace et al., 2003; Srivastava et al., 2012). Finally, the global distribution of evidence is patchy, with a lack of data in areas of high importance for reptile diversity, such as Southeast Asia, South America, and sub‐Saharan Africa (Roll et al., 2017).

The geographic gaps and biases that we found have also been reported by studies on reptile research more generally (Tingley et al., 2016; Tolley et al., 2016) and for other taxa (Christie et al., 2020, 2021; di Marco et al., 2017; Doi & Takahara, 2016; Roberts et al., 2016). Taking Africa as a specific example, although 16% of all terrestrial reptiles are found in Africa (Roll et al., 2017)—most likely an underestimate due to lack of sampling effort (Tingley et al., 2016)—only 5% of reptile studies were conducted in African nations, and many African nations had no studies at all (Figure 1). These geographic gaps also include some areas containing more threatened reptiles, such as Southeast Asia, West Africa, Madagascar, the northern Andes, and the Caribbean (Cox et al., 2022). However, overall countries with higher proportions of threatened reptile species did not have more studies, aligning with findings of Reboredo Segovia et al. (2020) that conservation research for mammals happens more in areas with more threatened mammal species. However, the magnitude of the effect of GDP per capita on the number of studies per country was relatively greater than the number of reptile species and the proportion of threatened species. This aligns with the findings of other studies that have identified biases in the conservation literature toward wealthier regions with higher research capacity (di Marco et al., 2017; Doi & Takahara, 2016; Roberts et al., 2016), which is typically positively correlated with GDP per capita. In other work, we have found that studies testing the effects of conservation actions on marine mammals are also highly skewed toward the Global North, most likely due to higher research capacity in these regions (Hordern et al., 2024). Taxonomic biases in conservation research are also well known (Christie et al., 2021; Donaldson et al., 2017; dos Santos et al., 2020), and reptiles are generally underrepresented in the research literature (Clark & May, 2002).

Testudines had significantly more studies per species than Squamata, which aligns with the findings of the IUCN Global Reptile Assessment (Cox et al., 2022). Squamata comprise 97% of all reptile species (Uetz et al., 2022), and although they had the highest overall number of studies, they were relatively underrepresented (Squamata: 0.035 studies per species; Testudines: 1.13 studies per species; Crocodilia: 1.5 studies per species); the majority of Squamata families had no studies at all (Figure 2). This is probably because of the much larger species diversity of Squamata, limited knowledge of many species relative to other orders (Oskyrko et al., 2024), and lack of known immediate threats compared to many species of Crocodilia and Testudines (Cox et al., 2022).

Our model predicting the number of studies per species suggests that species with fewer Wikipedia page views, that lack information on their venomousness, that are insular or endemic species, and not classified as least concern (in particular data‐deficient and not evaluated species) tend to be less studied. A general explanation for this may be that if there is less information on species (e.g., on distribution, ecology, and key threats), they are less likely to be studied, as conservationists are less aware of them and it is harder to test how those species respond to specific interventions. This may be a particular problem in the tropics where sampling effort is often less intensive (Hughes et al., 2021). The fact that we found no strong evidence that conservation‐relevant factors, such as ED or threatened status, were positively associated with the number of studies per species also aligns with findings for felid and canid mammal species (Tensen, 2018). We further found that factors potentially associated with extinction risk, such as insularity and endemism and body mass (Kier et al., 2009; Manes et al., 2021; Ripple et al., 2017), were not positively associated with the number of studies per species, and in fact, insular or endemic species were less likely to be studied (Hughes et al., 2021).

One explanation for these findings is that studies might be testing interventions on more common, non‐threatened species that are used as surrogates for more threatened, rarer, and thus potentially more difficult‐to‐study species. However, this seems unlikely because there are large species groups with limited or no evidence, meaning relevant evidence from closely related taxa is also likely to be lacking for many families, particularly Squamata families. As such, an alternative explanation is that research effort is being directed toward those species that are local to where capacity for conducting research is highest. For example, any reptile conservation research conducted in the United Kingdom will target one of only 6 species that are found there, none of which are globally threatened. A potential avenue for future research would be to consider the conservation status of a species at a national level.

It is also likely that certain taxonomic gaps and biases are directly related to geographic gaps and biases. For example, there were relatively few studies in Southeast Asia and so it is less surprising that this evidence synthesis (Sainsbury et al., 2021) found no studies testing actions on some of the endangered endemic species extant in the region, such as the earless monitor lizard (*Lanthanotus borneensis*) and the Chinese crocodile lizard (*Shinisaurus crocodilurus*) (Uetz et al., 2022). Other types of studies, such as on reptile husbandry, may exist for these species, but they were out of scope for Sainsbury et al. (2021), which excluded studies that only looked at reptile husbandry where no results on captive breeding were reported. Some of these gaps and biases could be addressed by expanding the evidence base on the effectiveness of actions to include studies published in other languages—only English‐language and certain German and Spanish journals were included in Conservation Evidence reptile searches. Since the publication of the Reptile Conservation Synopsis, a separate catalog of non‐English‐language studies (Conservation Evidence) testing actions has been added to the Conservation Evidence database, including searches of 316 journals (∼425,000 articles) published in 14 other languages (including additional Spanish and German journals) (Amano et al., 2021). These literature searches returned 44 studies of potential relevance to reptiles published in non‐English languages, although it is likely that only 30–40% of these would have been included following screening of full texts (e.g., due to lack of relevant data). There may be additional studies in other journals that were not covered by Sainsbury et al. (2021), particularly those with a focus on captive breeding and husbandry. Therefore, although the synopsis may have missed some non‐English‐language studies testing in‐scope conservation actions due to language bias (Amano et al., 2021), it is unlikely that this relatively small number would substantially alter the major patterns we found (Amano et al., 2021; Christie et al., 2021).

Another aspect to consider when interpreting our findings is funding, which is often directed toward the conservation of more popular or charismatic species (Joseph et al., 2011; Lundberg & Arponen, 2022). Several reptile species are widely considered as being charismatic due to a mixture of aesthetic and popular appeal (e.g., sea turtles, such as green and loggerhead turtles) and specific traits of interest, such as being venomous or large (e.g., some venomous snakes, komodo dragon)—although we did not find that venomous species were more studied than non‐venomous species. Indeed, species with a higher number of Wikipedia page views (as a proxy for popularity) tended to have a higher number of studies but were not necessarily any more threatened or evolutionarily distinct. Species rarely become popular due to their conservation need or perceived instrumental value (e.g., for ecosystem services) but instead may be born from marketing campaigns or purely aesthetic values (Ducarme et al., 2013). However, if popular species are also by chance highly threatened or can act as flagships for wider conservation goals, directing funding toward them could promote more cost‐effective conservation (McGowan et al., 2020). Although using flagship species to conserve reptiles seems an attractive approach to conservation, hotspots for turtles and lizards overlap poorly with other groups of tetrapods and so this approach may only have limited utility for other reptile taxa (Roll et al., 2017).

### Recommendations

Although there is a growing evidence base for reptile conservation that can help guide effective action, our results suggest that biodiversity conservation would benefit from a more strategic approach to determining which species and which actions are studied, as well as increasing the testing and reporting of the effects of conservation actions (Tingley et al., 2016). Moreover, a more strategic approach would benefit the conservation of all taxonomic groups, not just reptiles (Christie et al., 2020, 2021). To ensure practitioners have robust evidence to inform their conservation practice and policy, we hope our study can be the beginning of a movement to provide strategic guidance on what the gaps are in the global evidence base and what evidence needs should be prioritized for the next decade to improve the outcomes of conservation actions for reptiles. For example, we suggest prioritizing studies of threatened species, evolutionarily distinct and globally endangered species, and regions where there is a particular lack of evidence (and colocated with regions of high reptile diversity), such as South America, sub‐Saharan Africa, and Southeast Asia. In addition, there is the need for greater exploration of the transferability of the effects of conservation actions across contexts (e.g., geographies and taxa), for example, to determine whether one could efficiently focus effort on testing actions on certain species as a proxy for others that may be difficult to study (i.e., using cross‐taxon surrogacy for data‐deficient and not evaluated species). We therefore call on the conservation community to work together to coordinate the strategic testing and reporting of the effects of conservation actions to ensure the evidence needed to inform effective and efficient conservation globally can be generated and shared.

## Supporting information



Supplementary Materials.

Supplementary Materials.
