# Supplementary material for: Exploring gaps, biases, and research priorities in the evidence for reptile conservation actions
Source: Conserv Biol. 2025 May 31;39(5):e70073. doi: 10.1111/cobi.70073 (PMC12451487; doi:10.1111/cobi.70073)
Supplement: Supplementary file 2 — Supplementary Materials. [file COBI-39-e70073-s001.docx]

Supporting Information for:
Exploring the evidence base for reptile conservation actions: gaps, biases and research priorities

***​***

**Appendix S1: Data for species on which a study has quantitatively tested the effect of a conservation action, in descending order of the number of studies the species featured in. See file Appendix S1.**

**Appendix S2: Summary of all candidate models used for model selection derived from a global generalized linear model with the response variable of number of studies and the explanatory variables: IUCN Red List category, ED score, Wikipedia page views, taxonomic order, body mass, venomousness, and endemism. AICc refers to Akaike information criterion corrected for small sample sizes and ΔAICc refers to the difference in AICc between models.**

| Int. | Body mass scaled | ED scaled | Insular/ Endemic (yes/no) | Order | Wikipedia  page  views | IUCN Red  List category | Venomous (yes/no/ unknown) | df | Log Lik | AICc | Δ AICc | weight |
| --- | --- | --- | --- | --- | --- | --- | --- | --- | --- | --- | --- | --- |
| -2.312 | NA | NA | + | + | 2.0558 | + | + | 14 | -2419.31 | 4866.662 | 0 | 0.4691 |
| -2.3053 | NA | -0.0922 | + | + | 2.0512 | + | + | 15 | -2418.93 | 4867.9 | 1.2384 | 0.2525 |
| -2.3113 | 0.0114 | NA | + | + | 2.0538 | + | + | 15 | -2419.3 | 4868.655 | 1.9936 | 0.1731 |
| -2.3051 | 0.0057 | -0.0918 | + | + | 2.0502 | + | + | 16 | -2418.93 | 4869.903 | 3.2416 | 0.0928 |
| -2.3882 | NA | NA | NA | + | 2.1282 | + | + | 13 | -2424.71 | 4875.45 | 8.7877 | 0.0058 |
| -2.3804 | NA | -0.1007 | NA | + | 2.1221 | + | + | 14 | -2424.25 | 4876.542 | 9.8798 | 0.0034 |
| -2.3884 | -0.0042 | NA | NA | + | 2.129 | + | + | 14 | -2424.71 | 4877.453 | 10.7915 | 0.0021 |
| -2.3808 | -0.0102 | -0.1013 | NA | + | 2.1239 | + | + | 15 | -2424.25 | 4878.538 | 11.8766 | 0.0012 |
| -2.4684 | NA | NA | + | + | 2.1201 | + | NA | 12 | -2440.53 | 4905.092 | 38.4301 | 0 |
| -2.4682 | NA | 0.0383 | + | + | 2.1231 | + | NA | 13 | -2440.46 | 4906.961 | 40.2994 | 0 |
| -2.4681 | 0.0052 | NA | + | + | 2.1192 | + | NA | 13 | -2440.53 | 4907.095 | 40.4327 | 0 |
| -2.4678 | 0.0076 | 0.0388 | + | + | 2.1218 | + | NA | 14 | -2440.46 | 4908.961 | 42.2997 | 0 |
| -2.5312 | NA | NA | NA | + | 2.1977 | + | NA | 11 | -2445.28 | 4912.594 | 45.9318 | 0 |
| -2.5314 | NA | 0.0233 | NA | + | 2.1998 | + | NA | 12 | -2445.26 | 4914.548 | 47.886 | 0 |
| -2.5316 | -0.0102 | NA | NA | + | 2.1995 | + | NA | 12 | -2445.28 | 4914.589 | 47.9277 | 0 |
| -2.5317 | -0.0088 | 0.0229 | NA | + | 2.2014 | + | NA | 13 | -2445.26 | 4916.546 | 49.8844 | 0 |
| -2.2004 | 0.1797 | NA | + | NA | 2.8104 | + | + | 13 | -2460.92 | 4947.88 | 81.2185 | 0 |
| -2.2059 | NA | NA | + | NA | 2.898 | + | + | 12 | -2462.5 | 4949.031 | 82.3697 | 0 |
| -2.2007 | 0.1798 | 0.0046 | + | NA | 2.8104 | + | + | 14 | -2460.92 | 4949.884 | 83.2221 | 0 |
| -2.2057 | NA | -0.0032 | + | NA | 2.8979 | + | + | 13 | -2462.5 | 4951.036 | 84.3738 | 0 |
| -2.3115 | 0.1549 | NA | NA | NA | 3.0618 | + | + | 12 | -2473.48 | 4970.984 | 104.3219 | 0 |
| -2.3137 | NA | NA | NA | NA | 3.1328 | + | + | 11 | -2474.51 | 4971.054 | 104.392 | 0 |
| -2.3114 | 0.1549 | -0.0016 | NA | NA | 3.0617 | + | + | 13 | -2473.48 | 4972.989 | 106.3267 | 0 |
| -2.3132 | NA | -0.0063 | NA | NA | 3.1324 | + | + | 12 | -2474.51 | 4973.055 | 106.3932 | 0 |
| -2.5555 | NA | NA | + | + | 2.5044 | NA | + | 8 | -2488.28 | 4992.571 | 125.9097 | 0 |
| -2.554 | NA | -0.0245 | + | + | 2.5031 | NA | + | 9 | -2488.25 | 4994.523 | 127.8614 | 0 |
| -2.5554 | 0.0003 | NA | + | + | 2.5043 | NA | + | 9 | -2488.28 | 4994.575 | 127.9131 | 0 |
| -2.5541 | -0.0011 | -0.0245 | + | + | 2.5033 | NA | + | 10 | -2488.25 | 4996.527 | 129.8652 | 0 |
| -2.3982 | 0.1993 | 0.169 | + | NA | 2.957 | + | NA | 12 | -2486.4 | 4996.833 | 130.1707 | 0 |
| -2.3988 | 0.1969 | NA | + | NA | 2.9546 | + | NA | 11 | -2487.68 | 4997.381 | 130.7192 | 0 |
| -2.4064 | NA | 0.1625 | + | NA | 3.0603 | + | NA | 11 | -2488.29 | 4998.609 | 131.9474 | 0 |
| -2.4065 | NA | NA | + | NA | 3.0539 | + | NA | 10 | -2489.47 | 4998.968 | 132.3066 | 0 |
| -2.7153 | NA | NA | NA | + | 2.6598 | NA | + | 7 | -2500.7 | 5015.405 | 148.7429 | 0 |
| -2.7117 | NA | -0.0491 | NA | + | 2.6564 | NA | + | 8 | -2500.59 | 5017.201 | 150.5397 | 0 |
| -2.7165 | -0.0494 | NA | NA | + | 2.669 | NA | + | 8 | -2500.6 | 5017.223 | 150.5608 | 0 |
| -2.4936 | 0.1728 | 0.1534 | NA | NA | 3.211 | + | NA | 11 | -2497.74 | 5017.515 | 150.853 | 0 |
| -2.4929 | 0.1726 | NA | NA | NA | 3.2041 | + | NA | 10 | -2498.79 | 5017.603 | 150.9416 | 0 |
| -2.4969 | NA | NA | NA | NA | 3.2848 | + | NA | 9 | -2500.01 | 5018.039 | 151.3768 | 0 |
| -2.4977 | NA | 0.1501 | NA | NA | 3.2943 | + | NA | 10 | -2499.01 | 5018.039 | 151.3774 | 0 |
| -2.7128 | -0.0518 | -0.0512 | NA | + | 2.6659 | NA | + | 9 | -2500.49 | 5019.003 | 152.341 | 0 |
| -2.7234 | NA | NA | + | + | 2.5824 | NA | NA | 6 | -2511.93 | 5035.869 | 169.2075 | 0 |
| -2.7215 | NA | 0.1214 | + | + | 2.5925 | NA | NA | 7 | -2511.28 | 5036.577 | 169.9156 | 0 |
| -2.7237 | -0.0067 | NA | + | + | 2.5838 | NA | NA | 7 | -2511.93 | 5037.868 | 171.2066 | 0 |
| -2.7214 | 0.0004 | 0.1214 | + | + | 2.5925 | NA | NA | 8 | -2511.28 | 5038.58 | 171.9186 | 0 |
| -2.3946 | 0.1738 | NA | + | NA | 3.1892 | NA | + | 7 | -2516.94 | 5047.884 | 181.2227 | 0 |
| -2.3957 | NA | NA | + | NA | 3.296 | NA | + | 6 | -2518.38 | 5048.765 | 182.1034 | 0 |
| -2.4002 | 0.1742 | 0.0701 | + | NA | 3.1878 | NA | + | 8 | -2516.72 | 5049.463 | 182.8009 | 0 |
| -2.4011 | NA | 0.0661 | + | NA | 3.2959 | NA | + | 7 | -2518.19 | 5050.389 | 183.7276 | 0 |
| -2.8574 | NA | NA | NA | + | 2.7434 | NA | NA | 5 | -2522.78 | 5055.564 | 188.9019 | 0 |
| -2.8577 | NA | 0.0872 | NA | + | 2.7528 | NA | NA | 6 | -2522.45 | 5056.903 | 190.2411 | 0 |
| -2.8587 | -0.0538 | NA | NA | + | 2.7535 | NA | NA | 6 | -2522.67 | 5057.353 | 190.6915 | 0 |
| -2.8589 | -0.0499 | 0.0854 | NA | + | 2.762 | NA | NA | 7 | -2522.36 | 5058.721 | 192.0591 | 0 |
| -2.0985 | 0.4637 | NA | + | + | NA | + | + | 14 | -2522.83 | 5073.706 | 207.0441 | 0 |
| -2.5645 | NA | NA | NA | NA | 3.5101 | NA | + | 5 | -2532.36 | 5074.724 | 208.0622 | 0 |
| -2.5673 | 0.1241 | NA | NA | NA | 3.4432 | NA | + | 6 | -2531.72 | 5075.451 | 208.7888 | 0 |
| -2.0976 | 0.4643 | -0.0158 | + | + | NA | + | + | 15 | -2522.82 | 5075.691 | 209.0291 | 0 |
| -2.5682 | NA | 0.0391 | NA | NA | 3.5112 | NA | + | 6 | -2532.29 | 5076.594 | 209.932 | 0 |
| -2.5711 | 0.1237 | 0.0395 | NA | NA | 3.4441 | NA | + | 7 | -2531.65 | 5077.318 | 210.6566 | 0 |
| -2.1091 | NA | NA | + | + | NA | + | + | 13 | -2530.49 | 5087.006 | 220.3438 | 0 |
| -2.1102 | NA | 0.0191 | + | + | NA | + | + | 14 | -2530.47 | 5088.98 | 222.3186 | 0 |
| -2.1947 | 0.4861 | NA | NA | + | NA | + | + | 13 | -2532.29 | 5090.625 | 223.9631 | 0 |
| -2.1925 | 0.4874 | -0.0328 | NA | + | NA | + | + | 14 | -2532.25 | 5092.542 | 225.88 | 0 |
| -2.5931 | 0.1921 | 0.2485 | + | NA | 3.3679 | NA | NA | 6 | -2543.71 | 5099.424 | 232.7624 | 0 |
| -2.5966 | NA | 0.2462 | + | NA | 3.4948 | NA | NA | 5 | -2545.45 | 5100.908 | 234.2461 | 0 |
| -2.5924 | 0.1938 | NA | + | NA | 3.3754 | NA | NA | 5 | -2546.42 | 5102.854 | 236.1918 | 0 |
| -2.5956 | NA | NA | + | NA | 3.4996 | NA | NA | 4 | -2548.12 | 5104.237 | 237.5748 | 0 |
| -2.2088 | NA | NA | NA | + | NA | + | + | 12 | -2540.54 | 5105.111 | 238.4492 | 0 |
| -2.2091 | NA | 0.0055 | NA | + | NA | + | + | 13 | -2540.54 | 5107.114 | 240.4517 | 0 |
| -2.1532 | 0.4359 | NA | + | + | NA | + | NA | 12 | -2548.94 | 5121.908 | 255.246 | 0 |
| -2.1526 | 0.4338 | 0.0688 | + | + | NA | + | NA | 13 | -2548.74 | 5123.52 | 256.8581 | 0 |
| -2.7401 | NA | 0.2085 | NA | NA | 3.7026 | NA | NA | 4 | -2557.81 | 5123.616 | 256.9537 | 0 |
| -2.7418 | 0.1412 | 0.2072 | NA | NA | 3.6214 | NA | NA | 5 | -2556.98 | 5123.973 | 257.3109 | 0 |
| -2.7349 | NA | NA | NA | NA | 3.6987 | NA | NA | 3 | -2559.71 | 5125.421 | 258.7597 | 0 |
| -2.7366 | 0.1465 | NA | NA | NA | 3.6169 | NA | NA | 4 | -2558.87 | 5125.735 | 259.0733 | 0 |
| -2.1618 | NA | NA | + | + | NA | + | NA | 11 | -2555.72 | 5133.466 | 266.804 | 0 |
| -2.1608 | NA | 0.0978 | + | + | NA | + | NA | 12 | -2555.32 | 5134.674 | 268.0126 | 0 |
| -2.2364 | 0.4584 | NA | NA | + | NA | + | NA | 11 | -2559.7 | 5141.418 | 274.7566 | 0 |
| -2.2365 | 0.4571 | 0.0408 | NA | + | NA | + | NA | 12 | -2559.63 | 5143.286 | 276.6241 | 0 |
| -2.2472 | NA | NA | NA | + | NA | + | NA | 10 | -2567.02 | 5154.062 | 287.3998 | 0 |
| -2.2471 | NA | 0.0727 | NA | + | NA | + | NA | 11 | -2566.8 | 5155.628 | 288.9664 | 0 |
| -1.8122 | 1.4216 | NA | + | NA | NA | + | + | 12 | -2599.04 | 5222.101 | 355.4389 | 0 |
| -1.8159 | 1.3974 | 0.0715 | + | NA | NA | + | + | 13 | -2598.84 | 5223.72 | 357.058 | 0 |
| -2.3414 | 0.5072 | NA | + | + | NA | NA | + | 8 | -2609.69 | 5235.385 | 368.7228 | 0 |
| -2.3441 | 0.5046 | 0.0544 | + | + | NA | NA | + | 9 | -2609.57 | 5237.159 | 370.4972 | 0 |
| -2.3492 | NA | NA | + | + | NA | NA | + | 7 | -2617.46 | 5248.931 | 382.2693 | 0 |
| -2.3539 | NA | 0.0941 | + | + | NA | NA | + | 8 | -2617.11 | 5250.241 | 383.5794 | 0 |
| -1.9506 | 1.3777 | 0.2021 | + | NA | NA | + | NA | 11 | -2626.86 | 5275.755 | 409.0934 | 0 |
| -1.9557 | 1.6866 | NA | NA | NA | NA | + | + | 11 | -2627.2 | 5276.43 | 409.7684 | 0 |
| -1.9528 | 1.4519 | NA | + | NA | NA | + | NA | 10 | -2628.35 | 5276.725 | 410.0627 | 0 |
| -2.5545 | 0.4894 | NA | NA | + | NA | NA | + | 7 | -2632.13 | 5278.269 | 411.6077 | 0 |
| -1.9582 | 1.6697 | 0.0457 | NA | NA | NA | + | + | 12 | -2627.13 | 5278.283 | 411.6216 | 0 |
| -2.5555 | 0.4886 | 0.0164 | NA | + | NA | NA | + | 8 | -2632.12 | 5280.252 | 413.5904 | 0 |
| -2.4084 | 0.4722 | NA | + | + | NA | NA | NA | 6 | -2638.77 | 5289.554 | 422.892 | 0 |
| -2.4057 | 0.4662 | 0.1548 | + | + | NA | NA | NA | 7 | -2637.85 | 5289.71 | 423.0479 | 0 |
| -2.5607 | NA | NA | NA | + | NA | NA | + | 6 | -2639.45 | 5290.901 | 424.2396 | 0 |
| -2.5644 | NA | 0.06 | NA | + | NA | NA | + | 7 | -2639.31 | 5292.625 | 425.9633 | 0 |
| -2.411 | NA | 0.1878 | + | + | NA | NA | NA | 6 | -2644.31 | 5300.623 | 433.9611 | 0 |
| -2.4142 | NA | NA | + | + | NA | NA | NA | 5 | -2645.67 | 5301.35 | 434.6885 | 0 |
| -1.7599 | NA | 0.319 | + | NA | NA | + | + | 12 | -2639.09 | 5302.201 | 435.539 | 0 |
| -1.7368 | NA | NA | + | NA | NA | + | + | 11 | -2642.93 | 5307.882 | 441.2205 | 0 |
| -2.0775 | 1.7296 | NA | NA | NA | NA | + | NA | 9 | -2656.52 | 5331.054 | 464.3922 | 0 |
| -2.0766 | 1.663 | 0.1678 | NA | NA | NA | + | NA | 10 | -2655.54 | 5331.102 | 464.4399 | 0 |
| -2.5964 | 0.4579 | NA | NA | + | NA | NA | NA | 5 | -2662.7 | 5335.41 | 468.7482 | 0 |
| -2.5966 | 0.4535 | 0.0994 | NA | + | NA | NA | NA | 6 | -2662.33 | 5336.663 | 470.0017 | 0 |
| -2.6009 | NA | NA | NA | + | NA | NA | NA | 4 | -2669.11 | 5346.218 | 479.5558 | 0 |
| -2.6008 | NA | 0.136 | NA | + | NA | NA | NA | 5 | -2668.4 | 5346.804 | 480.1426 | 0 |
| -1.9041 | NA | 0.451 | + | NA | NA | + | NA | 10 | -2664.38 | 5348.784 | 482.1219 | 0 |
| -1.9645 | 1.7617 | NA | + | NA | NA | NA | + | 6 | -2671.24 | 5354.496 | 487.8344 | 0 |
| -1.9744 | 1.6909 | 0.1541 | + | NA | NA | NA | + | 7 | -2670.39 | 5354.791 | 488.1288 | 0 |
| -1.9028 | NA | NA | + | NA | NA | + | NA | 9 | -2671.73 | 5361.48 | 494.8178 | 0 |
| -1.927 | NA | 0.3924 | NA | NA | NA | + | + | 11 | -2677.8 | 5377.634 | 510.9718 | 0 |
| -1.907 | NA | NA | NA | NA | NA | + | + | 10 | -2683.08 | 5386.182 | 519.5206 | 0 |
| -2.1095 | 1.6839 | 0.309 | + | NA | NA | NA | NA | 5 | -2701.82 | 5413.636 | 546.9743 | 0 |
| -2.2024 | 1.9283 | NA | NA | NA | NA | NA | + | 5 | -2702.8 | 5415.615 | 548.953 | 0 |
| -2.2091 | 1.887 | 0.0808 | NA | NA | NA | NA | + | 6 | -2702.58 | 5417.166 | 550.504 | 0 |
| -2.1088 | 1.8444 | NA | + | NA | NA | NA | NA | 4 | -2705.12 | 5418.241 | 551.5791 | 0 |
| -2.0485 | NA | 0.5197 | NA | NA | NA | + | NA | 9 | -2702.62 | 5423.249 | 556.5874 | 0 |
| -2.0557 | NA | NA | NA | NA | NA | + | NA | 8 | -2711.48 | 5438.966 | 572.3039 | 0 |
| -1.7845 | NA | 0.5778 | + | NA | NA | NA | + | 6 | -2724.33 | 5460.667 | 594.0053 | 0 |
| -2.321 | 1.8793 | 0.2212 | NA | NA | NA | NA | NA | 4 | -2734.03 | 5476.064 | 609.4023 | 0 |
| -2.3157 | 2.0022 | NA | NA | NA | NA | NA | NA | 3 | -2735.64 | 5477.29 | 610.6282 | 0 |
| -1.682 | NA | NA | + | NA | NA | NA | + | 5 | -2737.07 | 5484.146 | 617.4845 | 0 |
| -1.9453 | NA | 0.7571 | + | NA | NA | NA | NA | 4 | -2753.25 | 5514.509 | 647.8474 | 0 |
| -2.0318 | NA | 0.5958 | NA | NA | NA | NA | + | 5 | -2758.8 | 5527.597 | 660.9352 | 0 |
| -1.9289 | NA | NA | NA | NA | NA | NA | + | 4 | -2771.52 | 5551.04 | 684.3785 | 0 |
| -1.8724 | NA | NA | + | NA | NA | NA | NA | 3 | -2774.54 | 5555.079 | 688.417 | 0 |
| -2.1628 | NA | 0.7581 | NA | NA | NA | NA | NA | 3 | -2786.6 | 5579.209 | 712.5473 | 0 |
| -2.0889 | NA | NA | NA | NA | NA | NA | NA | 2 | -2806.8 | 5617.608 | 750.9458 | 0 |

***​​***

**Appendix S3: Data for the top 100 reptile species that were most viewed on Wikipedia. ED score stands for Evolutionary Distinctiveness score (see Methods).**

| Rank | Binomial | Red List Category | Order | Family | ED | No. of studies | Wikipedia page views | Insular/ endemic (yes/no) | Venomous (yes, no, unknown) | Max. body mass (g) |
| --- | --- | --- | --- | --- | --- | --- | --- | --- | --- | --- |
| 1 | Varanus komodoensis | Vulnerable | Squamata | Varanidae | 7 | 1 | 7318871 | Yes | Unknown | 109647.8 |
| 2 | Dendroaspis polylepis | Least Concern | Squamata | Elapidae | 7 | 0 | 4399833 | No | Yes | 8213.6 |
| 3 | Ophiophagus hannah | Vulnerable | Squamata | Elapidae | 22 | 1 | 4169811 | No | Yes | 17723.3 |
| 4 | Crocodylus porosus | Least Concern | Crocodilia | Crocodylidae | 13 | 2 | 3954281 | No | No | 150000 |
| 5 | Agkistrodon piscivorus | Least Concern | Squamata | Viperidae | 6 | 1 | 2779029 | No | Yes | 3333 |
| 6 | Alligator mississippiensis | Least Concern | Crocodilia | Alligatoridae | 52 | 8 | 2451347 | No | No | 62000 |
| 7 | Crocodylus niloticus | Least Concern | Crocodilia | Crocodylidae | 9 | 4 | 2109870 | No | No | 750000 |
| 8 | Heloderma suspectum | Near Threatened | Squamata | Helodermatidae | 22 | 2 | 2100088 | No | Yes | 977.2 |
| 9 | Dermochelys coriacea | Vulnerable | Testudines | Dermochelyidae | 44 | 31 | 2067831 | No | No | 950000 |
| 10 | Pantherophis guttatus | Least Concern | Squamata | Colubridae | 3 | 3 | 1984895 | No | No | 543.7 |
| 11 | Boa constrictor | Least Concern | Squamata | Boidae | 23 | 0 | 1948355 | No | No | 35283.8 |
| 12 | Chelydra serpentina | Least Concern | Testudines | Chelydridae | 7 | 24 | 1933152 | No | No | 35000 |
| 13 | Crocodylus acutus | Vulnerable | Crocodilia | Crocodylidae | 7 | 3 | 1918477 | No | No | 539586 |
| 14 | Gavialis gangeticus | Critically Endangered | Crocodilia | Gavialidae | 32 | 2 | 1870975 | No | No | 806101 |
| 15 | Macrochelys temminckii | Vulnerable | Testudines | Chelydridae | 10 | 4 | 1853291 | No | No | 113000 |
| 16 | Oxyuranus microlepidotus | Least Concern | Squamata | Elapidae | 8 | 0 | 1829013 | No | Yes | 2290 |
| 17 | Python bivittatus | Vulnerable | Squamata | Pythonidae | 8 | 0 | 1735048 | No | No | 197681.2 |
| 18 | Malayopython reticulatus | Least Concern | Squamata | Pythonidae | 10 | 1 | 1692688 | No | No | 246603.9 |
| 19 | Pseudonaja textilis | Least Concern | Squamata | Elapidae | 6 | 3 | 1641546 | No | Yes | 1683.5 |
| 20 | Eunectes murinus | Least Concern | Squamata | Boidae | 4 | 1 | 1641355 | No | No | 345143.7 |
| 21 | Python regius | Least Concern | Squamata | Pythonidae | 12 | 2 | 1449264 | No | No | 1610.6 |
| 22 | Vipera berus | Least Concern | Squamata | Viperidae | 2 | 5 | 1435668 | No | Yes | 227.4 |
| 23 | Bungarus caeruleus | Least Concern | Squamata | Elapidae | 6 | 0 | 1383926 | No | Yes | 970.5 |
| 24 | Chelonia mydas | Endangered | Testudines | Cheloniidae | 22 | 49 | 1374237 | No | No | 230000 |
| 25 | Hemidactylus frenatus | Least Concern | Squamata | Gekkonidae | 14 | 0 | 1355227 | No | No | 6.3 |
| 26 | Naja naja | Least Concern | Squamata | Elapidae | 1 | 0 | 1324334 | No | Yes | 1683.5 |
| 27 | Lampropeltis triangulum | Least Concern | Squamata | Colubridae | 2.5 | 3 | 1250695 | No | No | 598.1 |
| 28 | Diadophis punctatus | Least Concern | Squamata | Colubridae | 21 | 6 | 1102642 | No | Yes | 118.5 |
| 29 | Notechis scutatus | Least Concern | Squamata | Elapidae | 5 | 2 | 1072373 | No | Yes | 1338.4 |
| 30 | Caretta caretta | Vulnerable | Testudines | Cheloniidae | 17 | 71 | 1071550 | No | No | 250000 |
| 31 | Dispholidus typus | Least Concern | Squamata | Colubridae | 10 | 0 | 1048775 | No | Yes | 558.9 |
| 32 | Daboia russelii | Least Concern | Squamata | Viperidae | 14 | 1 | 1031048 | No | Yes | 3122.3 |
| 33 | Chrysemys picta | Least Concern | Testudines | Emydidae | 6 | 23 | 1003537 | No | No | 2006 |
| 34 | Iguana iguana | Least Concern | Squamata | Iguanidae | 11 | 0 | 990506 | No | No | 8128.3 |
| 35 | Bothrops asper | Least Concern | Squamata | Viperidae | 5 | 0 | 988106 | No | Yes | 7499 |
| 36 | Varanus salvator | Least Concern | Squamata | Varanidae | 1 | 1 | 973474 | No | Unknown | 41686.9 |
| 37 | Natrix natrix | Least Concern | Squamata | Colubridae | 10 | 5 | 961450 | No | No | 2800.9 |
| 38 | Thamnophis sirtalis | Least Concern | Squamata | Colubridae | 8 | 16 | 932392 | No | Yes | 862.3 |
| 39 | Crocodylus palustris | Vulnerable | Crocodilia | Crocodylidae | 13 | 1 | 929480 | No | No | 450000 |
| 40 | Bothrops insularis | Critically Endangered | Squamata | Viperidae | 3 | 0 | 912344 | Yes | Yes | 843.7 |
| 41 | Crotalus horridus | Least Concern | Squamata | Viperidae | 9 | 5 | 859563 | No | Yes | 3333 |
| 42 | Centrochelys sulcata | Vulnerable | Testudines | Testudinidae | 22 | 0 | 855570 | No | No | 81000 |
| 43 | Bitis gabonica | Vulnerable | Squamata | Viperidae | 5 | 0 | 813432 | No | Yes | 2883 |
| 44 | Gekko gecko | Least Concern | Squamata | Gekkonidae | 9 | 1 | 805707 | No | No | 169.8 |
| 45 | Melanosuchus niger | Vulnerable | Crocodilia | Alligatoridae | 17 | 0 | 758927 | No | No | 883398 |
| 46 | Eretmochelys imbricata | Critically Endangered | Testudines | Cheloniidae | 19 | 17 | 754525 | No | No | 140000 |
| 47 | Pseudechis porphyriacus | Least Concern | Squamata | Elapidae | 16 | 0 | 742122 | No | Yes | 1338.4 |
| 48 | Plestiodon fasciatus | Least Concern | Squamata | Scincidae | 5 | 10 | 713729 | No | No | 13.8 |
| 49 | Amblyrhynchus cristatus | Vulnerable | Squamata | Iguanidae | 11 | 0 | 707248 | Yes | No | 7413.1 |
| 50 | Aldabrachelys gigantea | Vulnerable | Testudines | Testudinidae | 28 | 4 | 701074 | Yes | No | 117200 |
| 51 | Alligator sinensis | Critically Endangered | Crocodilia | Alligatoridae | 31 | 4 | 687493 | No | No | 84000 |
| 52 | Crotalus atrox | Least Concern | Squamata | Viperidae | 4 | 2 | 677641 | No | Yes | 6163.1 |
| 53 | Salvator merianae | Least Concern | Squamata | Teiidae | 14 | 0 | 671879 | No | No | 4466.8 |
| 54 | Drymarchon couperi | Least Concern | Squamata | Colubridae | 1 | 1 | 653265 | No | No | 1364.1 |
| 55 | Varanus bengalensis | Least Concern | Squamata | Varanidae | 4 | 0 | 638775 | No | Unknown | 18197 |
| 56 | Gopherus agassizii | Vulnerable | Testudines | Testudinidae | 5 | 18 | 622999 | No | No | 10956 |
| 57 | Crotalus adamanteus | Least Concern | Squamata | Viperidae | 6 | 0 | 611182 | No | Yes | 7630.7 |
| 58 | Lepidochelys olivacea | Vulnerable | Testudines | Cheloniidae | 16 | 22 | 604345 | No | No | 33000 |
| 59 | Eublepharis macularius | Least Concern | Squamata | Eublepharidae | 2 | 0 | 590209 | No | No | 114.8 |
| 60 | Dendroaspis angusticeps | Least Concern | Squamata | Elapidae | 6 | 0 | 579904 | No | Yes | 2290 |
| 61 | Boiga irregularis | Least Concern | Squamata | Colubridae | 6 | 1 | 554670 | No | Yes | 867.8 |
| 62 | Pantherophis obsoletus | Least Concern | Squamata | Colubridae | 2 | 6 | 540588 | No | No | 1281.4 |
| 63 | Lampropeltis californiae | Least Concern | Squamata | Colubridae | 0 | 0 | 540140 | No | No | 681.3 |
| 64 | Malaclemys terrapin | Vulnerable | Testudines | Emydidae | 10 | 15 | 532690 | No | No | 1599 |
| 65 | Pantherophis alleghaniensis | Least Concern | Squamata | Colubridae | 2 | 2 | 524676 | No | Unknown | 1281.4 |
| 66 | Ouroborus cataphractus | Least Concern | Squamata | Cordylidae | 24 | 0 | 515317 | No | No | 58.9 |
| 67 | Naja haje | Least Concern | Squamata | Elapidae | 2 | 0 | 512234 | No | Yes | 2516.8 |
| 68 | Sistrurus catenatus | Least Concern | Squamata | Viperidae | 11 | 7 | 484637 | No | Yes | 568 |
| 69 | Correlophus ciliatus | Vulnerable | Squamata | Diplodactylidae | 11 | 0 | 484000 | Yes | No | 45.7 |
| 70 | Oxyuranus scutellatus | Least Concern | Squamata | Elapidae | 8 | 0 | 459716 | No | Yes | 4665.5 |
| 71 | Trioceros jacksonii | Least Concern | Squamata | Chamaeleonidae | 10 | 0 | 454571 | No | No | 91.2 |
| 72 | Varanus giganteus | Least Concern | Squamata | Varanidae | 8 | 0 | 452586 | No | Unknown | 21379.6 |
| 73 | Bungarus fasciatus | Least Concern | Squamata | Elapidae | 14 | 0 | 444321 | No | Yes | 1777.1 |
| 74 | Morelia spilota | Least Concern | Squamata | Pythonidae | 3 | 2 | 440222 | No | No | 21710.2 |
| 75 | Opheodrys vernalis | Least Concern | Squamata | Colubridae | 4 | 1 | 423420 | No | No | 68.7 |
| 76 | Gopherus polyphemus | Vulnerable | Testudines | Testudinidae | 17 | 18 | 420699 | No | No | 10008 |
| 77 | Anolis sagrei | Least Concern | Squamata | Dactyloidae | 7 | 0 | 413989 | No | No | 8.7 |
| 78 | Thamnophis proximus | Least Concern | Squamata | Colubridae | 3 | 4 | 412919 | No | Yes | 562.3 |
| 79 | Testudo horsfieldii | Vulnerable | Testudines | Testudinidae | 22 | 2 | 406995 | No | No | 4123 |
| 80 | Crotalus cerastes | Least Concern | Squamata | Viperidae | 11 | 0 | 403739 | No | Yes | 313.8 |
| 81 | Heterodon platirhinos | Least Concern | Squamata | Colubridae | 9 | 2 | 391462 | No | Yes | 243.6 |
| 82 | Geochelone elegans | Vulnerable | Testudines | Testudinidae | 21 | 0 | 390389 | No | No | 9519 |
| 83 | Indotyphlops braminus | Least Concern | Squamata | Typhlopidae | 5 | 1 | 387654 | No | No | 2.1 |
| 84 | Acanthophis antarcticus | Least Concern | Squamata | Elapidae | 1 | 1 | 385344 | No | Yes | 252.3 |
| 85 | Crotalus scutulatus | Least Concern | Squamata | Viperidae | 6 | 0 | 381065 | No | Yes | 1387.5 |
| 86 | Apalone ferox | Least Concern | Testudines | Trionychidae | 12 | 1 | 379947 | No | No | 23463 |
| 87 | Hemidactylus turcicus | Least Concern | Squamata | Gekkonidae | 7 | 0 | 377396 | No | No | 5.8 |
| 88 | Ptyas mucosa | Least Concern | Squamata | Colubridae | 6 | 1 | 374560 | No | No | 3248.4 |
| 89 | Varanus niloticus | Least Concern | Squamata | Varanidae | 4 | 0 | 366335 | No | Unknown | 23988.3 |
| 90 | Lampropholis guichenoti | Least Concern | Squamata | Scincidae | 5 | 2 | 359924 | No | No | 2.6 |
| 91 | Thamnophis radix | Least Concern | Squamata | Colubridae | 1 | 2 | 354039 | No | Yes | 383 |
| 92 | Crocodylus johnstoni | Least Concern | Crocodilia | Crocodylidae | 14 | 0 | 352034 | No | No | 195000 |
| 93 | Tomistoma schlegelii | Vulnerable | Crocodilia | Crocodylidae | 51 | 0 | 350523 | No | No | 590000 |
| 94 | Moloch horridus | Least Concern | Squamata | Agamidae | 13 | 0 | 349710 | No | No | 61.7 |
| 95 | Echis carinatus | Least Concern | Squamata | Viperidae | 17 | 0 | 347990 | No | Yes | 272.3 |
| 96 | Bungarus multicinctus | Least Concern | Squamata | Elapidae | 2 | 0 | 347219 | No | Yes | 523.4 |
| 97 | Testudo hermanni | Near Threatened | Testudines | Testudinidae | 23 | 8 | 344596 | No | No | 7363 |
| 98 | Phrynosoma cornutum | Least Concern | Squamata | Phrynosomatidae | 20 | 2 | 339029 | No | No | 63.1 |
| 99 | Aspidoscelis neomexicanus | Least Concern | Squamata | Teiidae | 5 | 1 | 335730 | No | No | 18.6 |
| 100 | Rafetus swinhoei | Critically Endangered | Testudines | Trionychidae | 29 | 0 | 331102 | No | No | 78694 |
